# A Comprehensive Ubiquitous Healthcare Solution on an Android™ Mobile Device

**DOI:** 10.3390/s110706799

**Published:** 2011-06-29

**Authors:** Pei-Cheng Hii, Wan-Young Chung

**Affiliations:** 1 Department of Electronic Engineering, Graduate School, Pukyong National University, Busan 608-737, Korea; E-Mail: pearlyhii@yahoo.com; 2 Department of Electronic Engineering, College of Engineering, Pukyong National University, Busan 608-737, Korea

**Keywords:** personal healthcare, ECG monitoring, wireless sensor network, android smart phone, mobile barcode decoder

## Abstract

Provision of ubiquitous healthcare solutions which provide healthcare services at anytime anywhere has become more favorable nowadays due to the emphasis on healthcare awareness and also the growth of mobile wireless technologies. Following this approach, an Android™ smart phone device is proposed as a mobile monitoring terminal to observe and analyze ECG (electrocardiography) waveforms from wearable ECG devices in real time under the coverage of a wireless sensor network (WSN). The exploitation of WSN in healthcare is able to substitute the complicated wired technology, moving healthcare away from a fixed location setting. As an extension to the monitoring scheme, medicine care is taken into consideration by utilizing the mobile phone as a barcode decoder, to verify and assist out-patients in the medication administration process, providing a better and more comprehensive healthcare service.

## 1. Introduction

Chronic diseases are recognized as the leading cause of mortality in the World. According to statistics [1], among the top 10 leading causes of death in 2009 in South Korea, eight are chronic diseases, as shown in Table 1. Having experienced the loss of a beloved one due to a chronic disease such as heart diseases, hypertensive diseases and diabetes, people are now becoming more conscious about healthcare. Long term and quality medication treatment is necessary for chronic disease patients to ensure the disease is under control as chronic diseases are long-lasting and recurrent. Thus, the overall global healthcare costs are exploding as the public’s demand for better quality of healthcare increases. The consequence of this growing demand is a shortage of medical professionals and suitable medical infrastructures. Therefore, radical changes are needed to solve the problem.

Previously, healthcare was focused on institutional care and on curing diseases, which is diagnosis-based treatment only. Patients only approach medical professionals when they are not feeling well. However, constant monitoring, early detection and management of chronic diseases are important to avoid the occurrence of complications and risks. If the healthcare monitoring and management process moves from clinical-centric to patient-centric [2], be done at residential area by the patient him/herself, it would be a great solution to the resource shortage problems in hospitals. This will also greatly improve the patient’s medical knowledge as well as make one more alert to one self’s health status.

The rapid developments in the technologies, the ease of use and the falling cost of mobile devices have contributed to great changes in today’s lifestyle. During the past decade, the concept of ubiquitous coverage has expanded in various fields in the society due to the rapid development of wireless mobile and IT technologies. A lot of applications which were initially available at a fixed location only have been transformed into ubiquitous applications, to be used wirelessly and flexibly at anytime and anywhere. For example, the capability to watch a television program and listen to songs on a mobile phone, compared to the older days where these former entertainment were available at fixed locations with power supply availability. The same trend has been observed in the medical field. Over the years, most telemedicine and healthcare related societies and authorities have been concerned with the merging of wireless technologies and healthcare to overcome the poor mobility of PC desktop-based monitoring systems. Thus, the possibility to monitor vital signs and biomedical signals using a mobile device is no longer an unachievable dream and task.

The increased number of chronic disease patients and the recent technological advances has inspired the idea of this paper, where wireless technologies are applied to help patients improve their situation. In this paper, a comprehensive ubiquitous healthcare solution which includes a real time ECG (electrocardiography) monitoring and analyzing system based on an Android mobile device and also provides medicine care assistance is proposed. WSN (wireless sensor network) technology is applied in this system to transmit the ECG data wirelessly from the patient’s body to an Android smart phone device. In order to achieve maximum mobility and flexibility in ubiquitous healthcare, the design and the size of the sensor node used are taken into consideration during the hardware implementation. As for medicine care assistance, barcode technology is applied to assist out-patients in medication administration, by capturing and decoding the barcode on medicines using the Android smart phone’s embedded camera. This reduces the occurrence of medical errors caused by consumption of the wrong medicine which is often life threatening. The idea of this proposal can drive healthcare provision out of hospitals and into the home environment by utilizing a mobile phone, with benefits in medicine, health and social care. It also reduces the hassles, queues and crowds in hospital as well as providing more healthcares services and focus to patients who are seriously and urgently in need of such services.

The remainder of this paper is organized as follows. The background and related works which inspired the idea of developing a comprehensive ubiquitous healthcare solution are described in Section 2. Section 3 presents the system design of the ubiquitous healthcare system and the implementation of the methods used in obtaining, handling and processing the sensory data from the biomedical device on the human’s body, followed by descriptions of experimental results in Section 4. Lastly, Section 5 contains some conclusions.

## 2. Background and Related Works

Ubiquitous healthcare applications may include disease-diagnosis devices, monitoring systems, and even healthcare information systems. In our approach, ECG monitoring is the main focus. ECG is a graphic recording of the heart’s electrical activity [3]. In an ECG test, the electrical impulses that occur when the heart beats are recorded in a waveform graph. ECG monitoring is an efficient and important clinical technique in healthcare as information from an ECG test can be used to discover heart diseases, determine the rate and regularity of heart beats as well as the size and position of chambers, and also to evaluate the effects of drugs or specialized devices used to regulate the heart. Since ECG is the core reference for doctors in the diagnosis and medication process, there are many types of commercialized ECG mobile monitoring applications available in the market today. Examples include Spyder wireless ECG monitoring [4] and wireless pulse/ECG watches [5].

In ubiquitous healthcare, wireless data communication technologies such as WSN and Bluetooth^®^ are adopted to transmit the data while mobile devices are used as the monitoring terminal. However, most market-available ubiquitous healthcare applications have adopted the Bluetooth^®^ technology. Spyder wireless ECG monitoring and wireless pulse/ECG watch are examples of Bluetooth^®^ communication-based mobile healthcare applications. Nevertheless, several limitations and problems are reported with the adoption of Bluetooth^®^ in ubiquitous healthcare communications such as the power hungry needs. WSN with the IEEE 802.15.4 Zigbee radio protocol which is able to overcome the problems seems to be more favorable for remote healthcare wireless communications. A WSN equipped with an IEEE 802.15.4 Zigbee radio protocol is preferable to Bluetooth^®^ with an IEEE 802.15.1 radio protocol due to its faster, more flexible and scalable networking features. The most important factor is that it consumes less energy, processing and memory resources [6].

In WSN, each sensor has wireless communication capability and some level of intelligence for signal processing and networking of the data. Other than direct transmission, a spatially distributed sensor has the capability to route the data using a multi-hop routing protocol by going through a few sensor nodes to a gateway sensor node known as a base station node. This provides better connectivity than the Bluetooth^®^ technology which only supports transmission up to a certain maximum range. Thus, WSN is adopted in this proposed ubiquitous healthcare application. A WSN is easier to deploy and it is scalable. A sensor node’s self organizing and automatic calibrating characteristics enable a single node to be added to or removed easily from a network. A network is reconfigured with dramatically less complexity and costs compared to wired networks as no fixed installation is needed. A WSN’s unobtrusiveness increases the patient’s acceptance in term of its portability. In short, WSNs and healthcare are a perfect match to bring healthcare from the hospital or medical center to one’s own home.

Along with the advances in communication technology, mobile communication devices can now provide efficient and convenient services such as remote information interchange and resource access through mobile devices so that users can work ubiquitously [7]. With the astronomical growth of the mobile phone ownership rate, mobile healthcare supported by mobile and wireless technologies no longer seems as costly and non-essential instead it is a cost-effective care solution with better overall health outcomes. A mobile device in ubiquitous healthcare must be an ultra compact, low cost, light-weight and low power consumption unit to achieve the optimal outcome.

In our work, an Android™-based smart phone is considered as the monitoring terminal due to its smart functions and computer-like features compared to the old conventional phones which are used mainly for calling and texting. Compared to other smart phone operation systems, the Android™ unit has many advantages, such as openness, all applications are equal, there are no boundaries between applications, and it achieves a fast and convenient development [8]. Furthermore, the Android™ smart phone has been identified as one of the most popular smart phones in the market.

## 3. System Design and Implementation

Medical resources are very precious. Normally hospitalized all-day-long care is only applied to patients who are in critical condition and need urgent medical treatment. Out-patients with chronic diseases that need only intensive care are not advised to occupy a hospital bed, but instead they need intensive home care to ensure their diseases are under control. Therefore, self-monitoring, self-management, mobility and flexibility are the key concepts for success of the implementation of a ubiquitous healthcare system for out-patients with chronic diseases. A ubiquitous healthcare system must be a robust, reliable and convenient application, so that patients can do around the clock monitoring and go around without any restrictions.

**Figure 1.**
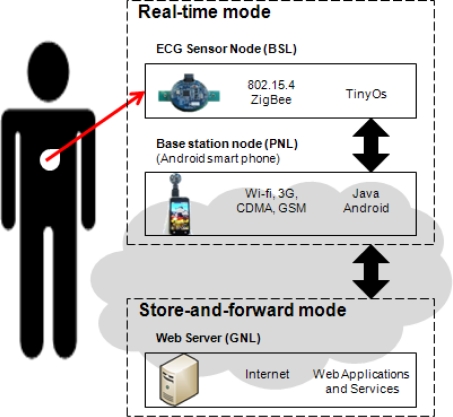
System design of real time ECG monitoring and analyzing on an Android™ mobile device.

The proposed system is mainly for ECG monitoring and heart rate estimation, which is useful in the detection of the underlying heart conditions of individuals and the rehabilitation of patients recovering from recent heart attacks. Figure 1 presents the system design of the proposed real time ECG monitoring and analyzing on an Android™ mobile device with hardware examples, communication protocol, and software implementation. There are three architecture layers in the proposed idea: the BSL (body sensor layer), PNL (personal network layer) and GNL (global network layer). The first layer, BSL, consists of ECG sensor nodes worn on the patient’s body. The ECG sensor node in the BSL communicates with the PNL which consists of a base station node and an Android™ smart phone exploiting the IEEE 802.15.4 Zigbee based communication protocol, to measure and transmit the real-time quality ECG signal wirelessly from ECG devices on the patient’s body to the mobile device for display and analysis. A smart phone with a base station node in PNL acts as a higher level data and communication coordinator, and allows for user-interaction with the body sensor network. An Android™ mobile device with wireless networking capabilities has the ability to communicate with a higher application services layer, the web server at GNL, with any mobile network provided by the telecommunications service provider, such as CDMA or GSM, 3G networks and Wi-Fi services. This enables the data transmission and communication between patients and doctors who are at a distant location.

In short, the proposed ubiquitous healthcare system is divided into two modes as shown in Figure 1, the real-time mode and the store-and-forward mode. In real time, the ECG signals are available to be viewed and monitored on the smart phone device immediately after the vital sign acquisition from the sensor node and the base station node. A patient is able to capture his heart condition through the real time signal presentation and analyze the performance immediately. In the store-and-forward mode, the mobile phone transfers data such as the medical history and the summary reports to the web server side for further comprehensive analysis. The data is stored and it can be accessed and retrieved at a later time as well.

### 3.1. Deployment of Sensor Nodes in WSN

**Figure 2.**
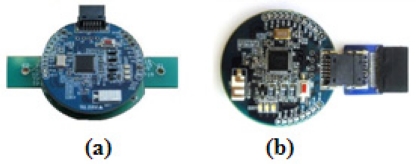
Sensor nodes: (**a**) Sensor node on ECG module; (**b**) Wireless dongle on smart phone.

**Figure 3.**
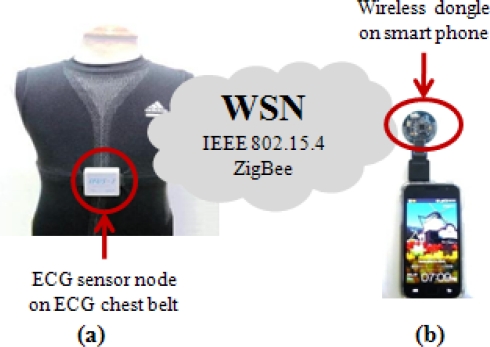
Deployment of sensor nodes in WSN: (**a**) Wearable health shirt embedded with ECG devices; (**b**) Wireless dongle on smart phone device.

The deployment of sensor nodes in a WSN is mainly for setting up a wireless network environment for ECG data transmission between the BSL and PNL. In this module, a compact, light-weight and small ECG sensor node as shown in Figure 2(a) is implanted in a body-worn ECG device, namely the wearable health shirt shown in Figure 3(a). This wearable health shirt with an ECG chest belt and ECG sensor node is able to obtain the ECG vital signs from the human’s body. This shirt can be easily applied to a person’s chest. The patients will not feel uncomfortably as it is just like wearing a normal shirt with no restrictions on their daily activities. A tiny base station node, known as the wireless dongle [9] and shown in Figure 2(b) is modified to be attached to a smart phone as shown in Figure 3(b) to receive the ECG vital signs from the wearable health shirt. The wireless dongle is connected to the smart phone through a customized sensor node-to-mobile phone RS-232 serial communication interface.

Both the ECG sensor node and the base station node have a serial port interface that provides bidirectional communication at 15,200 Kbps which allows them to connect to the ECG module internally. These sensor nodes are specially designed for ubiquitous healthcare applications. The size of the mote is small −4 × 4 × 0.2 cm. These sensor nodes are featured with a Texas Instruments MSP430F1611 ultra low power microcontroller, equipped with an internal voltage and ECG sensor together with a CC2420 RF transceiver. The specifications of the sensor nodes are listed in Table 2. A sensor node attached to the ECG module is connected to a rechargeable battery to allow for the continuous ECG data transmission and monitoring. The wireless dongle is modified to be attached to the mobile device according to one of ubiquitous healthcare’s basic concepts, flexibility, where monitoring process can be done anytime without any obstacles, especially with regard to the the battery exhaustion problem. This modified node attached to the smart phone is different from the other sensor modules which normally have a battery power supply module attached. It utilizes the power supply directly from the battery of the mobile phone. The user does not need to worry about battery usage at the base station as long as the mobile phone is functioning.

**Figure 4.**
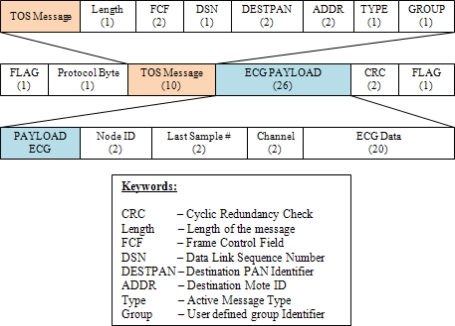
Packet format of sensor data.

The ECG data obtained by the sensor node from the wearable smart shirt is translated into sensory data and then transmitted by the sensor nodes over the network to the wireless dongle, the base station node at the smart phone. The sensor nodes are built on top of the TinyOS’s embedded C based programming platform. Figure 4 shows the packet format of the sensory data in the implementation. This packet format has the same encoding as that of the HDLC protocol. Data is organized into frames and sent across WSN to its destination. The packet format contains a flag field to identify the beginning and end of a packet frame, a protocol byte, followed by 10-byte of TinyOS command message, 26-byte of ECG payload and a 2-byte CRC. A TinyOS command message consists of length, frame control field, data link sequence number, destination identifier, message type and a group identifier. For ECG data, 20 bytes are allocated for 10 data with 2 bytes each.

### 3.2. ECG Monitoring System Implementation

**Figure 5.**
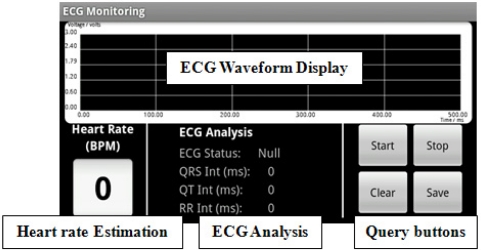
GUI of ECG monitoring system.

The received sensor data is further processed and interpreted by the ECG monitoring system running on the Android™ mobile device. In the ECG monitoring system, various algorithms are combined and implemented as mobile application software with the Java Android™ language to handle all the query processes in the WSN. The query processes handle the communication between sensor nodes and the ECG device, detect and differentiate the ECG sensory data, interpret, analyze and manipulate the sensory data packet, and then, they proceed to display the data graphically on a mobile screen in real time. Figure 5 shows the data visualization of the GUI of the ECG monitoring system. It includes an ECG waveform display, ECG waveform analysis, real time heart rate estimation and also queries buttons to handle the process to initiate and end a monitoring activity.

**Figure 6.**
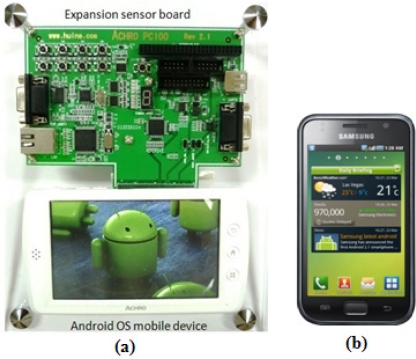
Android™ mobile devices: (**a**) AchroHD (Huins Inc. Korea); (**b**) Galaxy S (Samsung Electronics Ltd., Korea).

The ECG monitoring system was developed and tested on an Android™ mobile platform development kit [10] as shown in Figure 6(a), running an ARM processor (S5PC100X—ARMCORTEX A8). This development kit comes with a sensor expansion board and an Android™-based smart phone-alike mobile device. Through the serial port, the sensor expansion board is able to connect to different types of hardware devices and various development testing can be performed on it. The specifications of the development kit are similar to the specifications of Galaxy S in Figure 6(b), a market available Android™ based operating system. Both the AchroHD development kit and the Samsung Galaxy S running on Android™ OS version 2.1 were used to test the implementation of our approach.

#### 3.2.1. Data Aggregation Process

**Figure 7.**
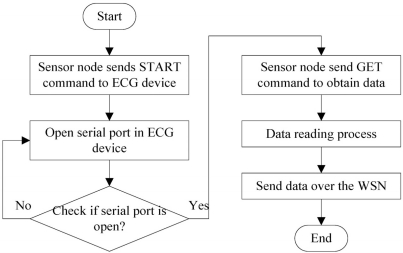
Communication between the sensor node and the ECG device.

In order to obtain the ECG sensory data accurately from the ECG device, the communication between the sensor nodes and the ECG device is of primary importance. Figure 7 shows the flow chart of the communication between the sensor nodes and the ECG device. To start a communication, a user sends a START mode to the sensor nodes through the query button. The START mode will open the serial port in the ECG device to initialize the data aggregation process. When the serial port is open, it indicates that the ECG device is ready to receive commands from the sensor node and that it is ready for transmission. A sensor node then sends a GET mode to obtain the ECG data from the ECG device. Once the reading process is done, a sensor node will communicate with the base station node in the network and transmit the sensory information back to the base station at the smart phone.

#### 3.2.2. Data Extraction and Manipulation Process

**Figure 8.**
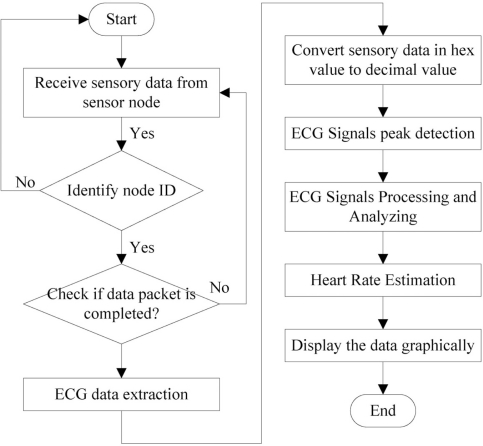
Data manipulation process.

Upon receiving the data packet from the ECG device, the packet is processed and useful data is extracted. An appropriate way to handle and manipulate the ECG data is vital because this is intimately related to data quality. Figure 8 shows the flow chart of the data manipulation process. A sensor node’s node ID is identified first when the data is received to ensure that the aggregated data is from the correct sensor source. Then, the received data is scanned through to ensure the data packet is a complete packet. The 20-byte of ECG data are extracted. The ECG data is converted to a decimal value and presented in waveform format on mobile phone. The ECG data presented in waveform is for better visualization and better understanding of the heart condition, a beneficial feature for both doctors and patients.

To perform precise ECG data analysis on a resource-limited mobile phone at real time, an accurate and simple technique is required instead of complicated techniques which involve more processing memory. In our approach, the QRS detection algorithm by Tompkins [11] is adopted for the detection of the QRS peak in the ECG waveform which can be used for further analysis. Information such as QRS interval time, QT interval time and RR interval time can be obtained from the detection of QRS peaks. This information is useful for pathophysiological indications of ECG. As an example, a shortened QT interval indicates hypercalcemia where as a prolonged QT interval indicates hypocalcemia.

### 3.3. Personalized Medicine Care Assistance

Smart phones have built-in cameras that can take pictures of interesting events but those cameras can also act as scanners. There are numbers of researchers [12,13] who have discussed the possibility of decoding a barcode with a mobile phone’s built-in camera. This idea is widely applied in many industrial areas as well as healthcare. Applications include asset management and patient administration process. In [14], the utilization of barcode and mobile phones for blinds and visually impaired people to identify objects by decoding the barcode to a URL and directs the phone’s browser to fetch an audio file from the web that contains a verbal description of that particular object was proposed. The possibility to decode a barcode with a mobile phone’s built-in camera inspired the idea to include personalized medicine care assistance into the ECG monitoring system to provide a more comprehensive ubiquitous healthcare solution.

The design of personalized medicine care assistance is to help chronic disease out-patients in their daily medication administration processes. According to a landmark study on medical errors conducted by the United State Institute of Medicine [15], medication errors and ADR (adverse drug reactions) are the most common cases among all medical errors. Most of these errors are nonetheless preventable [16]. Out-patients with chronic diseases normally need to undergo long term medical treatment and medication process and they are required to take many types of medicine daily to control their diseases. A common cause of medical errors includes irregular medicine in-take due to the patient’s busy or erratic lifestyles, complicated in-take due to many medicines and doses taken by the patient, ADR caused by un-reconciled prescriptions obtained from different sources, lack of knowledge about proper use of medicines, lack of consultation with healthcare provides when confusion arises and lack of monitoring mechanisms to keep track of a patient’s medicine in-take. Thus, out-patient medication administration has been identified as the most error prone procedure. Various medicine in-take reminder or support systems are introduced to help this group of patients. Wedjat [17], a mobile phone based medicine in-take reminder and monitor, is one such example.

**Figure 9.**
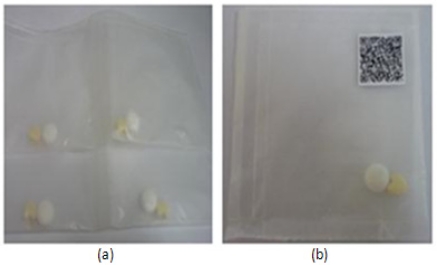
Sample medicine packs in Korea: (**a**) Medicine packs; (**b**) Medicine pack with barcode.

Personalized medicine care assistance is designed to assist patients in the medication administration process to avoid taking the wrong medicines. In Korea, all medicines that are to be consumed at once are packed into one pack as shown in Figure 9 when a patient receives the medicines at the drug store. For chronic disease out-patients, where too many daily medicines in-takes are necessary, patients might mix up and get confused of which medicines to take at particular time without a proper guidance. Based on this, barcode technology is applied where a personalized barcode image is generated and attached on the medicine packs. Every time before the medicines in-take, patient can use his smart phone’s camera which has higher pixels to capture the barcode image clearly, decode the image with embedded decoding algorithm, to get little guidance and verify the medicines before medicines in-takes, to ensure the right medicines are taken. Wrong medication in-take can be a killer.

#### 3.3.1. Personalized QR Barcode Generator

**Figure 10.**
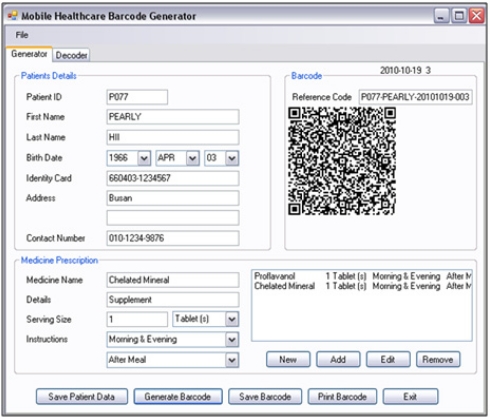
Personalized QR barcode generator in ubiquitous healthcare system.

**Figure 11.**
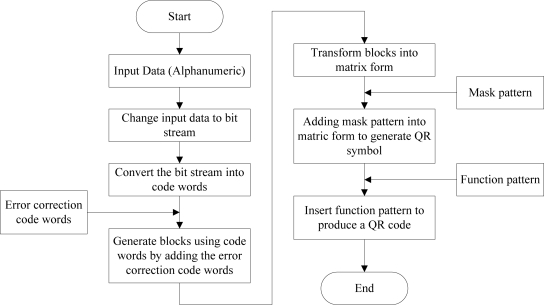
Data encoding process.

Nowadays, most smart phones are come with a free barcode decoding application. This is beneficial for smart phone users as they can fully utilize it without any need for additional hardware devices and charges. In this implementation, a personalized QR (Quick-Response) barcode generator is designed and implemented as shown in Figure 10, to meet the needs of ubiquitous healthcare system’s users, and provides a comprehensive healthcare services to the users. This personalized QR barcode generator is implemented in the C# programming language. Information such as patient name, patient ID, functions of the drugs, in-take instructions, dosage amount and expired date are included in the barcode for the patients’ reference. Other than generate the barcode, a copy of the input details will be saved at the web server database for future reference. The QR barcode is adopted as it has larger and sufficient data capacity to encode all the information needed if compare to the 1 dimension parallel barcode. Alphanumeric data are encoded into QR barcode through encoding steps shown in Figure 11. To implement the personalized generator, QRode library [18], a .NET component is used to do the data encoding and generate a QR barcode. The QRCode library provides a function to encode the content into a QR code image which can be saved in JPEG, GIF, PNG or Bitmap formats, and also a function to decode a QR code image. The QR barcode generated can be printed out easily and can be attached to the medicine packs easily.

## 4. Experimental Results and Discussions

The algorithms implemented are tested by setting up a physical real time monitoring test bed to test the ability of data collection from an ECG device and from the sensor nodes as well as to evaluate the data transmission over the WSN. Obtained sensory data is manipulated, processed, analyzed and displayed graphically on the smart phone screen. The ability to decode the personalized barcode with smart phone’s built-in camera is tested as well.

### 4.1. Real Time ECG Monitoring Module

**Figure 12.**
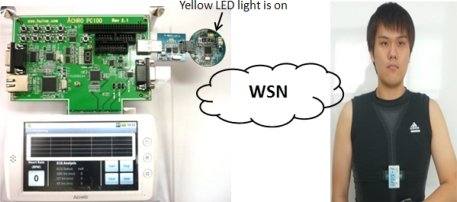
Setup of ECG real time monitoring environment.

In the real time ECG monitoring module, a wearable health shirt is worn on a human body. The ability to provide a real time ECG signal from the wearable health shirt and the capability to capture the signal and present it on an Android™ mobile device is observed. Figure 12 shows the example of a patient wearing the body-fitted ECG health shirt. The figure shows that the sensor node embedded on the wearable health shirt is small and inobtrusive. The sensor nodes on the ECG health shirt and on the mobile device establish a WSN. The yellow light on the wireless dongle indicates that the wireless connection is established and that data transmission is available. When the Start button on the mobile screen is pressed, the wireless dongle attached to the mobile device is ready to receive and process the ECG data packet from the ECG device.

**Figure 13.**
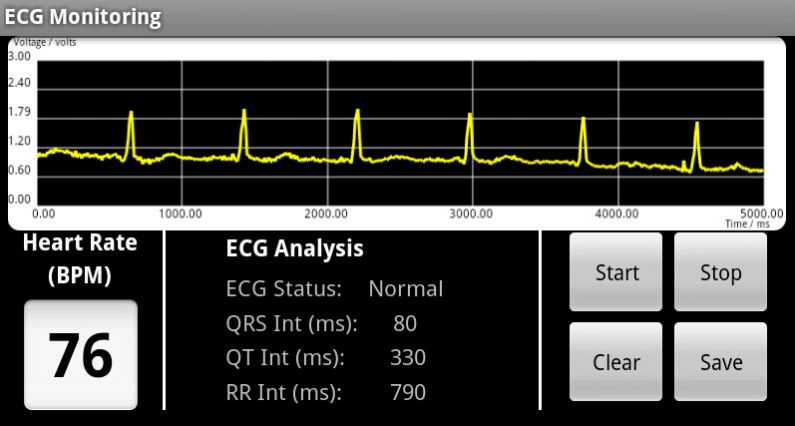
Screen capture of the real time ECG monitoring system.

Figure 13 shows a screen capture of the real time ECG monitoring screen on the mobile phone. The ECG vital signs are displayed graphically on the screen and further analysis of ECG vital signs is performed. QRS peaks are detected and the interval time between the waveforms, the QT and RR intervals, are calculated. This information is useful for doctors in the diagnosis and treatment process as information such as the heart rate can be estimated, the condition of heart can be determined, and any symptoms of heart attack can be detected. The data sampling rate in this system is 100 Hz, which means 10 data packets are sent within 1 second where 1 data packet consists of 10 ECG data, so 100 ECG data are sent in 1 second. The waveform display on this monitoring module is designed to be able to display up to 500 data in 5,000 milliseconds. The large screen (400 × 800 pixels) of the Android™ mobile device provides a clear visualization graphic for the user, compared to our previous research work [9], which used the older generation of mobile phones, the bar type phone.

### 4.2. Personalized Medication Care Module

**Figure 14.**
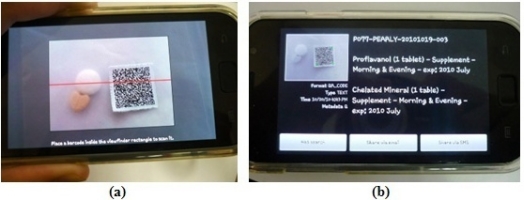
Personalized medicine care assistance: (**a**) Screen capture of mobile barcode decoder trying to decode the personalized QR barcode; (**b**) Display of decoded data on mobile phone.

The capability to decode our self-generated and personalized QR barcode was tested. Figure 14(a) shows the screen capture of the mobile barcode decoder on an Android™ smart phone device trying to decode a barcode image. The decoded data are shown in Figure 14(b). This mobile barcode decoder is available free of charge. By utilizing this mobile barcode decoder, it is proved that the personally generated barcode image earlier is encoded correctly and that it can be decoded easily as well. The extracted information is used as guidance for the patient in his medication administration process.

The QR barcode is adopted here as the data capacity is large and sufficient to encode a long piece of alphanumeric text on a little barcode image. The decoding time of the QR barcode is short and accurate regardless of how many characters are encoded. Table 3 shows a comparison of the average barcode decoding time between QR barcode with 170 characters and 23 characters from a real time experiment with the Samsung Galaxy S Android™ smart phone. The results show that decoding a barcode with 170 characters and 23 characters takes almost the same time. Thus, the QR barcode is suitable for use in ubiquitous healthcare applications. Another benefit of the QR barcode is that it can be decoded in every way or position regardless of how the barcode is placed with respect to the scanner.

## 5. Conclusions and Future Work

Ubiquitous healthcare solutions on Android™ mobile devices are believed to have a significant impact in bringing heart rate management and ECG monitoring to individuals and patients in everyday life. The development of technology has greatly increased our diagnostic power. These developments have been widely reported, creating a widespread acceptance in both society and patients. This has increased the public expectations not only for high technology healthcare but also for rapid and unrestricted high quality healthcare services. Chronic diseases can be effectively controlled if they are regularly monitored with proper medication cares and guides.

WSNs are expected to fulfill the unrestricted conditions of healthcare applications, hoping to reduce the mortality rate caused by chronic diseases. Wirelessly transmitting the ECG signal in a WSN can reduce the hassle of traditional wired ECG machines, provide a clean and stable ECG signal for real time heart rhythm analysis and achieve self monitoring, mobility and flexibility. Other than moving healthcare from clinical-centric to patient-centric, this idea would also move the healthcare from treatment to prevention. The early detection of diseases might give a recovery chance to the patient. The rise in global expenditures, shortage of medical staffs and equipment problems and growing incidences of chronic illness can be solved as well.

With additional personalized medicine care assistance in a healthcare system, a more comprehensive and affordable healthcare solution is provided to the patient, assisting the patient in the medication administration process, without the need of any extra hardware devices and costs. As a conclusion, the proposed solution is easy to be applied with only an ultra ECG wearable device embedded with a sensor node and an Android™ smart phone device.

At this moment, this Android™ based ubiquitous healthcare system is only able to monitor ECG vital signs in real time. In the future, more health parameters such as blood pressure, blood glucose level, and body temperature are considered to be included in this system, to provide more health information and a more precise monitoring scheme. An alarm system can also be included in the system as well to activate an alarm sound sending warning messages wirelessly to a doctor’s mobile phone when an event occurs. Using GPS capability, the location of the patient will be easily tracked then if rescue is needed.

## Figures and Tables

**Table 1.** The leading causes of deaths in South Korea (2009).

**Rank**	**Cause of death**	**No. of deaths**	**%**	**Death rate**
1	Malignant neoplasms	69, 780	28.3	140.5
2	Cerebrovascular diseases	25, 838	10.5	54.0
3	Heart diseases	22, 347	9.0	45.0
4	Suicides	15, 413	6.2	31.0
5	Diabetes	9, 757	4.0	19.6
6	Transport accidents	7, 147	2.9	14.4
7	Chronic lower respiratory diseases	6, 914	2.8	13.9
8	Liver diseases	6, 868	2.8	13.8
9	Pneumonia	6, 324	2.6	12.7
10	Hypertensive diseases	4, 749	1.9	9.6

(Unit: per 100,000 population, person, %)

**Table 2.** Specifications of the sensor node.

**Components**	**Descriptions**
Microcontroller	MSP430F1611
RF Transceiver CC2420	Sensitivity:12 bits Transceiver rate:250 Kbps Rx current:18.8 mA Tx current:17.4 mA
RF range	≈100 m
Size (cm)	4 × 4 × 0.2
Power	2.5 V–4.0 V

**Table 3.** Comparison of average barcode decoding time.

**Comparison of average barcode decoding time**		
Length of encoded data	170 characters	23 characters
Average decoding time required	1.41 s	1.39 s
